# Non-equilibrium supramolecular polymerization

**DOI:** 10.1039/c7cs00121e

**Published:** 2017-03-28

**Authors:** Alessandro Sorrenti, Jorge Leira-Iglesias, Albert J. Markvoort, Tom F. A. de Greef, Thomas M. Hermans

**Affiliations:** a University of Strasbourg , CNRS , ISIS UMR 7006 , F-67000 Strasbourg , France . Email: hermans@unistra.fr; b Computational Biology Group and Institute for Complex Molecular Systems , Eindhoven University of Technology , P.O. Box 513 , 5600 MB Eindhoven , The Netherlands . Email: t.f.a.d.greef@tue.nl

## Abstract

Supramolecular polymerization has been traditionally focused on the thermodynamic equilibrium state, where one-dimensional assemblies reside at the global minimum of the Gibbs free energy. The pathway and rate to reach the equilibrium state are irrelevant, and the resulting assemblies remain unchanged over time. In the past decade, the focus has shifted to kinetically trapped (non-dissipative non-equilibrium) structures that heavily depend on the method of preparation (*i.e.*, pathway complexity), and where the assembly rates are of key importance. Kinetic models have greatly improved our understanding of competing pathways, and shown how to steer supramolecular polymerization in the desired direction (*i.e.*, pathway selection). The most recent innovation in the field relies on energy or mass input that is dissipated to keep the system away from the thermodynamic equilibrium (or from other non-dissipative states). This tutorial review aims to provide the reader with a set of tools to identify different types of self-assembled states that have been explored so far. In particular, we aim to clarify the often unclear use of the term “non-equilibrium self-assembly” by subdividing systems into dissipative, and non-dissipative non-equilibrium states. Examples are given for each of the states, with a focus on non-dissipative non-equilibrium states found in one-dimensional supramolecular polymerization.

## Abstract 2

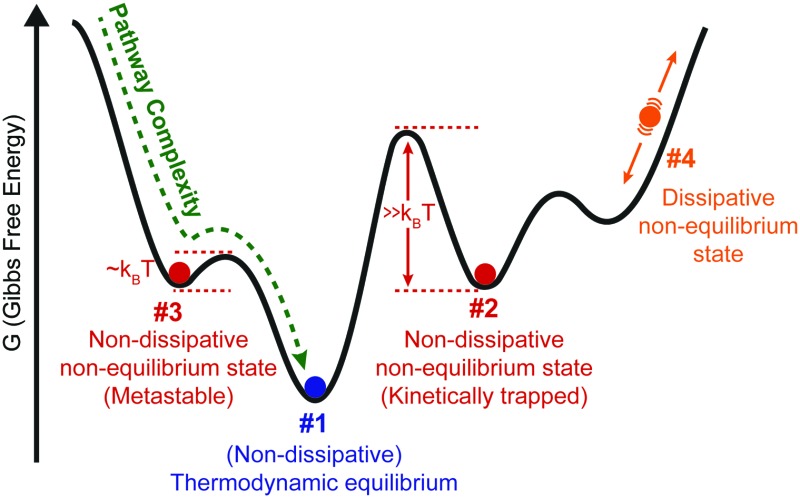
Supramolecular polymers can reside in four distinct thermodynamic states. The preparation protocol and mechanistic insights allow to identify each one of them. Going beyond equilibrium polymerization is an exciting new direction in the field of supramolecular chemistry.

##

Key learning points

(1) There are different thermodynamic states in supramolecular polymerization: equilibrium, non-dissipative non-equilibrium, and dissipative non-equilibrium.

(2) Supramolecular polymerization kinetics are of key importance in all thermodynamic states, including at equilibrium.

(3) Mechanistic insights using kinetic models are needed to fully understand pathway complexity.

(4) Control over polymerization kinetics can lead to pathway selection, where different structures are formed depending on the preparation protocol.

(5) Distinct supramolecular structures can exist at the same experimental conditions when different assembly pathways are present.

## Introduction

Molecular self-assembly is centrally important in living systems, where supramolecular structures resulting from the self-assembly of two or more molecular components are used to perform complex functions like translation, replication, signalling and cellular transport. Some remarkable examples are the double-stranded DNA, ribosomes, cell membranes and G-proteins.^1^ Over the past decades, supramolecular chemists have designed building-blocks that self-assemble into well-defined supramolecular architectures according to the chemical information encoded in their molecular structure.^2^–^5^ This has resulted in a plethora of supramolecular systems, many of which have been engineered to respond to a variety of stimuli (*e.g.* pH, light, ionic strength, *etc.*).

Recently, Fujita and co-workers reported, for example, on a monodisperse sphere formed by the incredible number of 144 self-assembled components (48 palladium ions and 96 bent ligands).^6^ Supramolecular polymers present a particularly well-studied subclass of structures in supramolecular chemistry, where monomers form one-dimensional aggregates through reversible and highly directional secondary interactions.^4^,^7^,^8^

**Fig. 1:**
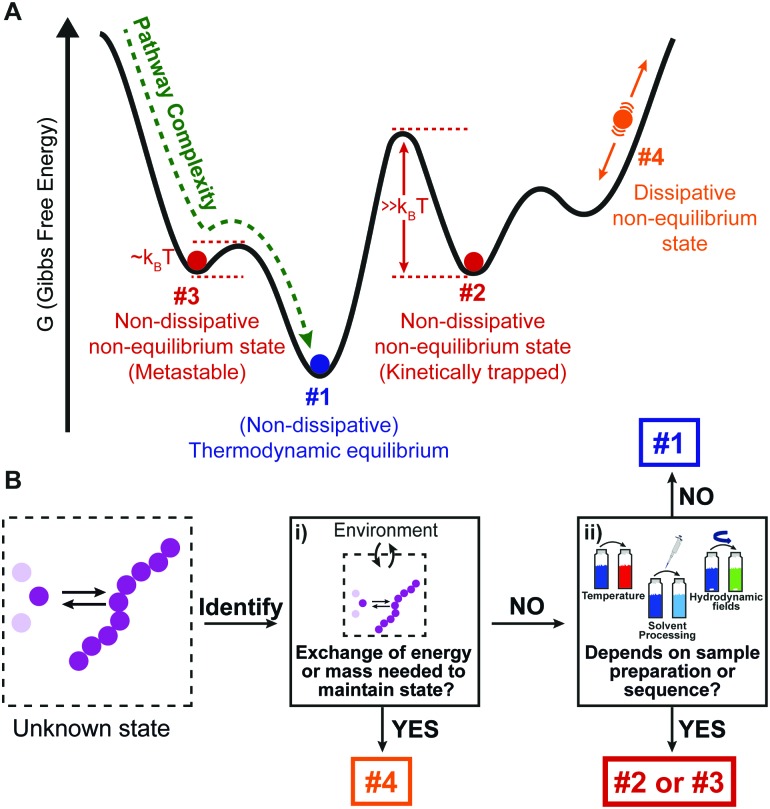
Identifying the different thermodynamic states in supramolecular self-assembly. (A) Schematic Gibbs free energy landscape. (B) Decision tree to identify the different states shown in (A).

Their unique mechanical properties and versatility have enabled new applications in supramolecular electronics, and biomedical devices for regenerative medicine.^4^ Different mechanisms of polymerization, *e.g.*, isodesmic, cooperative, or anti-cooperative (see Section 1), have been described so far, but in general the focus has been on the equilibrium state (#1 in Fig. 1A).^7^,^8^ The latter is reached when fully reversible non-covalent interactions are involved, which enable the system to explore different configurations (*i.e.* walking along the energy landscape), and to find the most stable one, by self-repairing possible defects.^5^ On the other hand, when strong non-covalent interactions come into play, the self-assembly process is often governed by the kinetics of the system, which may lead to kinetically trapped or metastable states (*i.e.*, non-dissipative non-equilibrium states, #2 or #3 in Fig. 1A), instead of the aggregate state corresponding to thermodynamic equilibrium. In the case of states #2 or #3, the outcome of the aggregation process, for example in terms of nanoscale morphology, becomes strongly dependent on the experimental procedures and preparation protocols, as a result of non-linear phenomena such as nucleation and multiple competitive growth pathways (*vide infra*). In recent years, the increased insight into kinetics and pathway complexity has provided exquisite control over aggregate morphology and molecular organization. In other words, the desired aggregation pathway can be rationally selected by suitable preparation methodologies, leading to assemblies with different targeted features starting from the same building-blocks (see Section 2). The latter is crucial to develop supramolecular materials with optimized functional properties.

Biological systems use molecular chaperones, not only to aid in protein folding, but also to control the self-assembly of supramolecular polymers such as flagellae (*i.e.*, long filaments that are involved in cell motility).^9^ The major filament of a bacterial flagella, for example, consists of 20 000 flagellin monomers, which are polymerized at a precisely defined location. Specific export chaperones bind to the flagellin monomers to prevent pre-mature polymerization. In particular, the chaperones help to avoid kinetically trapped aggregates that might form along wrong self-assembly pathways by shielding the monomers from incorrect interactions that may occur on their way to the polymerization site. In artificial supramolecular polymerization, however, chaperones have not been invented yet.

In addition, living systems use another class of supramolecular polymers that continuously consume energy to stay in dissipative non-equilibrium states (#4 in Fig. 1A). In this case, the energy provided by a chemical fuel (*e.g.* guanosine triphosphate GTP for microtubules, and adenosine triphosphate ATP for actin fibers) allows spatiotemporal control over the self-assembly, enabling the cell to perform complex functions such as cell division, motility, and intracellular transport.^1^ Recently, there is a growing interest among supramolecular chemists for synthetic dissipative systems, although this is still a major challenge and only a few examples have been reported so far.^10^–^13^

What we have shown so far is that there are four different thermodynamic states where supramolecular polymerization can take place (#1, #2, #3, and #4, Fig. 1A). However, a clear and universally used definition of these different states is essentially lacking, which can be problematic for new researchers entering the field. Here we provide a simple classification and description of the different states, and how to identify them using a decision tree:

• *Thermodynamic equilibrium*: the system resides in the global minimum of the free energy landscape (state #1 in Fig. 1A). No external energy inputs are required to maintain the system, and on average no changes are observed in time. Note that dissipation, usually in the form of heat release, does occur during the downhill self-assembly process from the monomers to the assembly. Once the equilibrium is reached, however, no further dissipation occurs.^14^ Equilibrium structures are still dynamic, meaning that the monomers continuously exchange with the solution, and their reversibility is crucial for the system to find and remain in its most stable configuration.^5^ It has to be stressed that, for a given molecular design (chemical information), the shape of the energy landscape depends on parameters such as temperature, solvent composition and salt concentrations. As a result, the morphology of the most stable aggregate (*i.e.*, the position of the global minimum) can change for different sets of these parameters (see Section 1). Nearby free energy minima—often observed in experiment as polymorphic structures—can also be populated according to the Boltzmann distribution if the activation barrier is low.^15^

• *Non-dissipative non-equilibrium states* (kinetically trapped or metastable states): the self-assembled system is confined in a local minimum of the energy landscape (states #2 and #3 in Fig. 1A). Its time evolution depends on the shape of the energy landscape around the minimum, and two situations can be envisaged. If the energy barrier for a pathway leading to the thermodynamic equilibrium is low enough, that is, on the same order of magnitude as *k*_B_*T* (where *k*_B_ is the Boltzmann constant, and *T* temperature), the system will slowly relax to a more stable structure. Such a system is in a so-called metastable state (#3 in Fig. 1A). Note that multiple metastable configurations can exist along the pathway to the global minimum. On the other hand, when the energy barrier is much higher than *k*_B_*T*, the system will remain captured in the local minimum for a period much longer than the experimental observation. This state is commonly referred to as a kinetically trapped state (#2 in Fig. 1A). In the latter case, suitable experimental procedures have to be undertaken to “help” the system to escape its trap (see Section 2).

• *Dissipative non-equilibrium state*: dissipative self-assembled systems require a constant influx of energy or matter (*e.g.*, a chemical fuel or light), and removal of waste products, to be kept steadily in a dissipative non-equilibrium state (state #4 in Fig. 1A). If the energy supply stops, the system relaxes spontaneously to the thermodynamic state or to a non-dissipative non-equilibrium state encountered on the way. The terminology of “dissipative structures” as formulated by Prigogine, refers to emergent structures or patterns that are formed on length scales much larger than the individual molecules, the latter of which at equilibrium do not form such structures or patterns. Instabilities occurring far from equilibrium, such as those due to reaction–diffusion phenomena, can lead to dissipative structures even on the mm scale, that is, far beyond the length scale of typical intermolecular interactions (*e.g.*, hydrogen bonding, ionic interactions, π–π stacking, *etc.*).^16^ In contrast, in supramolecular chemistry well-ordered structures often already exist in non-dissipative states, and to this day it is unclear how dissipation in self-assembly has to be related to dissipative structures in the Prigogine-sense. What is clear, is that dissipative self-assembly is a very exciting new direction^10^–^13^ where challenges, such as obtaining non-equilibrium steady states (NESS) or oscillations, are abundant. We refer to the review by van Esch, Eelkema, and Boekhoven in this same issue for more in depth information on dissipative non-equilibrium states.

### Decision tree to identify thermodynamic states

In Fig. 1B, we provide a decision tree to assign a given self-assembled system to one of the states discussed above.

(i) Does the system need to exchange energy and/or matter with the environment to maintain its structure over prolonged times?

YES: it is a *dissipative non-equilibrium state* (#4, Fig. 1).

NO: 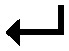


(ii) Does the obtained supramolecular structure depend on the preparation protocol, that is, on parameters such as rate of cooling, solvent processing, order of addition, *etc.*?

YES: it is a *non-dissipative non-equilibrium state*, either *metastable* (#3, Fig. 1), if it evolves slowly with time to a more stable state, or *kinetically trapped* if it remains indefinitely (#2, Fig. 1).

NO: the same structure is obtained independently of the history of the sample. It is the thermodynamic equilibrium (#1, Fig. 1).

In what follows below we will first focus on one-dimensional supramolecular polymerization, and the importance of kinetic processes (Section 1). After that, we provide practical methods on how to control non-dissipative non-equilibrium states in the laboratory (Section 2).

## 1. Non-dissipative states (#1, #2, #3): a mechanistic understanding

**Fig. 2:**
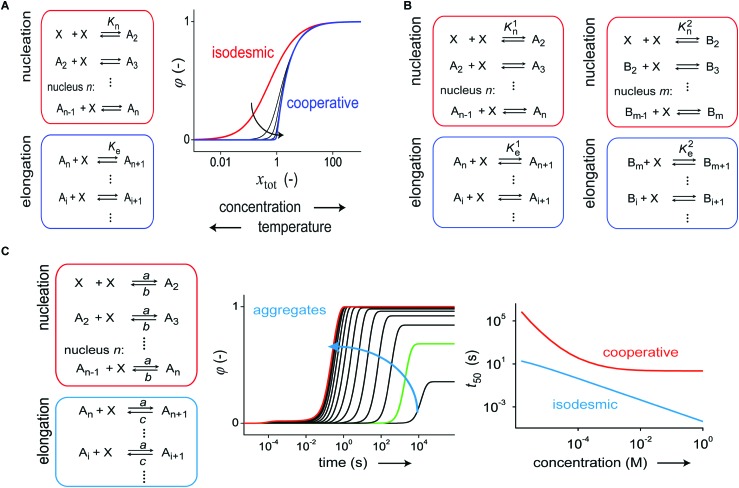
(A) Schematic representation of a one-dimensional supramolecular polymerization in which aggregates, A_i_, grow exclusively by monomer addition. When the association equilibrium constants are equal (*K*_n_ = *K*_e_) aggregate growth occurs *via* an isodesmic self-assembly mechanism, and when *K*_n_ < *K*_e_, a cooperative nucleation–elongation mechanism is followed. The exact mechanism has a profound influence on the fraction of aggregation, *φ*(–), as a function of temperature or concentration. (B) Schematic representation of a supramolecular polymerization involving two aggregate states, A and B which are coupled by monomer X. In both pathways, aggregate growth can occur *via* several potential mechanisms *i.e.* isodesmic (*K*_n_ = *K*_e_), nucleation–elongation (*K*_n_ < *K*_e_) or anticooperative (*K*_n_ > *K*_e_). Because of the different temperature dependency of the equilibrium constants, different aggregate states become dominant in different temperature regimes. (C) Kinetic nucleation–elongation model of a single-pathway supramolecular polymerization based on monomer association and dissociation. Simulations performed (*a* = 10^4^ M^–1^ s^–1^, *b* = 1 s^–1^, *c* = 0.01 s^–1^, *n* = 5) reveal faster growth at higher total monomer concentration. Isodesmic and cooperative growth can be distinguished by plotting *t*_50_, the time at which *φ*(–) is equal to 0.5 as a function of total monomer concentration. Reproduced from ref. 18 with permission from American Chemical Society, copyright 2014.

At thermodynamic equilibrium, the indefinite self-assembly of small molecules into one-dimensional nanostructures can be expressed as a sequence of reversible monomer addition steps in which each step is described by a decrease/increase in Gibbs free energy.^7^ In an isodesmic supramolecular polymerization mechanism, the Gibbs free energy of all individual steps are equivalent and thus independent of the length of the aggregate. Such a supramolecular polymerization can be characterized by a single equilibrium constant that depends on the chemical structure of the monomer, the solvent, and temperature. Cooperative (or nucleated) supramolecular polymerization is characterized by the formation of a thermodynamically unfavorable oligomer (nucleus) followed by energetically favored elongations steps. As a result, this mechanism is characterized by two equilibrium constants that describe reversible monomer addition to pre-nucleus oligomers and post-nucleus polymers. These mechanisms can be distinguished by measuring the fraction of aggregation using spectroscopic techniques as a function of temperature or total concentration under equilibrium conditions (Fig. 2A).^17^,^18^

In an increasing number of examples, supramolecular polymerizations result in multiple aggregate states at thermodynamic equilibrium representing different aggregate morphologies. This typically occurs when self-assembly is probed at different temperatures, solvent compositions or salt concentrations resulting in complex supramolecular energy landscapes.^19^ While one aggregate can be the most thermodynamically stable morphology at one temperature, solvent composition or salt concentration, a different morphology becomes the most stable aggregate under different conditions.

Recently, Meijer and de Greef analysed a thermodynamic model which contains two self-assembly pathways, representing two different aggregate morphologies that compete for the same monomer (Fig. 2B).^20^ Temperature dependent simulations revealed that the model is able to describe competition between aggregate morphologies which can have a profound influence on the shape of melting curves.

In an impressive study, Stupp and co-workers^19^ investigated the energy landscape of a peptide amphiphile (PA) with the sequence V_3_A_3_K_3_ conjugated to an alkyl chain at the N-terminus, which self-assembles in water into nanofibers. Because of the presence of charged residues in the peptide, the self-assembly is sensitive to the ionic strength. A detailed analysis revealed that at low ionic strength, the PA monomers self-assemble into short fibrils with a predominant random coil structure. However, at higher salt concentrations, the PA fibrils are much longer and have a beta-sheet structure indicating that the self-assembly of PA is characterized by multiple aggregate morphologies that are stable under different conditions. In short, equilibrium structures depend on the experimental conditions at the thermodynamic equilibrium, but not on how that state it was reached.

While thermodynamic characterization of supramolecular assemblies is now a common routine, the kinetic characterization of supramolecular polymerizations has lagged behind. Similar to thermodynamic models, the kinetics of supramolecular polymerizations can be described by a sequence of monomer association and dissociation steps. For an isodesmic supramolecular polymerization each step in the growth can be modeled with two rate constants which describe addition and dissociation of a monomer to a growing chain. By solving the corresponding system of coupled ordinary differential equations (ODE), the fraction of aggregation *φ*(*t*), and mean length as a function of time can be computed.^17^,^20^ Also for nucleated supramolecular polymerizations, kinetic models based on aggregate growth by monomer addition and dissociation have been analyzed.^20^,^21^ These models employ different association and dissociation rate constants for pre-nucleus oligomers and post-nucleus aggregates although often the association rate constant is assumed to be independent of the length of the aggregate (Fig. 2C). Analysis of such models reveals that the time evolution of the fraction of aggregation is characterized by the appearance of a lag time at low concentrations, which is described by a quadratic relation.

The isodesmic and nucleated polymerization mechanisms can be discriminated by measuring concentration-dependent kinetic curves and evaluating *t*_50_, the time at which the fraction of aggregation *φ*(*t*) is equal to 0.5, as a function of concentration (Fig. 2C).^18^,^21^,^22^ Next to monomer association and dissociation, nucleated supramolecular polymerizations can also proceed *via* fragmentation and coagulation reactions, which can modulate the assembly kinetics. While the influence of these reactions strongly depends on the cooperativity, recent simulations using realistic rate constants have revealed only a minor influence on the time-evolution of the fraction of aggregation.^21^ However, the addition of fragmentation and coagulation reactions strongly influenced the time-evolution of the mean aggregate length resulting in a faster equilibration.

Instead of monitoring the time-dependent evolution of the fraction of aggregation *φ*(*t*), supramolecular chemists typically characterize the kinetics of supramolecular polymerizations by acquiring different heating and cooling curves. By measuring the fraction of aggregation at various heating and cooling rates (°C s^–1^), one can detect hysteresis in some cases. Hysteresis is a kinetic phenomenon and implies failure of opposing reactions to equilibrate on the timescale of the heating/cooling experiment.^7^ Typically, hysteresis occurs when there is a large kinetic barrier in the assembly or disassembly pathway (*i.e.*, #2, Fig. 1A), for example, when aggregate growth is dominated by homogeneous nucleation. It is important to realize that the observation of hysteresis in supramolecular polymerization is by itself not an indication of the presence of multiple aggregation pathways as hysteresis can already be observed for supramolecular polymerizations that result in a single aggregate state. However, when multiple aggregate pathways are present, hysteresis, quantified by the area between the heating and cooling curve, can be enhanced.^23^

**Fig. 3:**
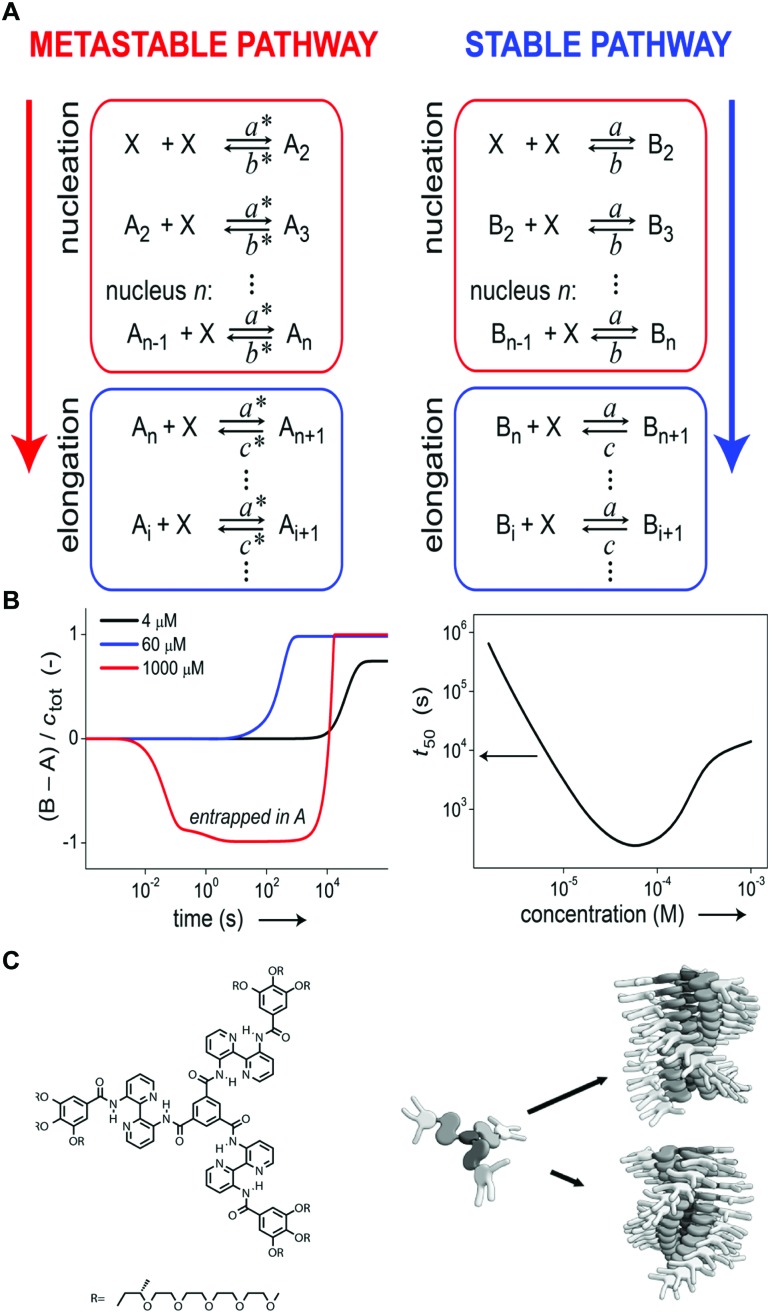
(A) Kinetic model, based on monomer association and dissociation, of a supramolecular polymerization consisting of two coupled nucleation–elongation pathways representing two different aggregate states, A and B. The model employs a total of six rate constants. (B) When one pathway is under kinetic control, the formation of thermodynamically stable A-type aggregates starting from the monomer is slowed down by sequestration of monomers in kinetically controlled B-type aggregates. This effect has a profound influence on the *t*_50_*vs.* concentration curves. Reproduced from ref. 18 with permission from American Chemical Society, copyright 2014. (C) Structure of the bipyridine-extended 1,3,5-benzenetricarboxamide discotic molecule equipped with chiral oligo(ethyleneglycol) side chains and schematic representation of the equilibrium between the two different aggregate states. Reproduced from ref. 20 with permission from American Chemical Society, copyright 2015.

In 2012, Korevaar *et al.* analyzed the supramolecular polymerization kinetics of *S*-chiral oligo(*p*-phenylenevinylene) (*S*-OPV) into helical aggregates using stopped-flow CD spectroscopy.^24^ Previous studies revealed that *S*-OPV self-assembles into a left-handed helical aggregate in apolar solvents such as MCH under thermodynamically controlled conditions. However, the kinetic data revealed that when aggregation is commenced from the monomeric state, less-stable, right-handed helical off-pathway aggregates are initially formed which are later converted to more stable left-handed assemblies. That is, off-pathway metastable species are formed (#3, Fig. 1A), which over time relax to the equilibrium state (#1, Fig. 1A). In order to describe their kinetic data, the authors formulated a kinetic model consisting of two parallel operating, indefinite self-assembly pathways that compete for the same monomer (Fig. 3A). An analysis of the data with the ODE model revealed that aggregate growth in both the kinetically (off-pathway) and thermodynamically (on-pathway) controlled pathways occurs *via* a nucleation–elongation mechanism. In addition, by studying the *t*_50_ as a function of concentration, the influence of the off-pathway aggregates was also corroborated. While a single pathway nucleation–elongation mechanism is characterized by a decrease of *t*_50_ for increasing concentrations followed by a plateau at high concentrations, the addition of the kinetically controlled pathway results in an inverted dependence of *t*_50_ at high concentrations (Fig. 3B).^22^,^24^ The original kinetic model developed by Korevaar and co-workers to study pathway complexity and kinetic control in supramolecular polymerization is based on aggregate growth by monomer association and dissociation events. Recently, Markvoort *et al.* analyzed the effect of fragmentation and coagulation reactions and found that the presence of these reactions may dramatically delay the formation of thermodynamically stable on-pathway aggregates.^21^

In many cases, supramolecular chemists use different solvent combinations to induce aggregation or to disassemble an existing aggregate (see Section 2). To this end, Meijer and de Greef recently studied the disassembly kinetics of helical aggregates based on *S*-OPV using chloroform as a denaturant.^25^ By explicitly incorporating the effect of the two solvents in their kinetic model, the authors could rationalize the disassembly kinetics as a function of the volume fraction of denaturant.

While the two-pathway kinetic model can be used to gain insight into kinetically controlled supramolecular polymerization, it can also be employed to probe conversion between two different aggregate states. To this end, van der Zwaag *et al.* recently investigated the interconversion mechanism of two different aggregate states in the supramolecular polymerization of a bipyridine-extended 1,3,5-benzenetricarboxamide building block (Fig. 3C).^20^ Spectroscopic analysis of the aggregation mechanism under steady-state conditions revealed the presence of two aggregate states at different temperatures. Kinetic experiments using temperature-jump spectroscopy and subsequent analysis with the two-pathway ODE model showed that the two aggregate states interconvert *via* the free monomer. Furthermore, the analysis revealed that the low temperature aggregate state assembled *via* an isodesmic mechanism while the high temperature aggregate state assembled *via* an anti-cooperative mechanism. This shows once more that the same molecule at two different temperatures can lead to two distinct equilibrium states, each with a distinct assembly mechanism and aggregate morphology.

The previous examples on kinetic control in supramolecular polymerization typically employ only a single component. In 2013, Korevaar *et al.* investigated the co-assembly kinetics of *S*-OPV and *R*-OPV monomers into helical aggregates.^26^ To investigate their co-assembly, majority rules experiments were conducted, which assess the capability of the major enantiomer to direct its chirality by forcing all aggregates in the system to take over its preferred helicity, so-called “majority rules”. When the two enantiomers were mixed at room temperature no majority rules effect was observed. However, when the mixture was heated above the elongation temperature and slowly cooled, chiral amplification was observed. In addition, the authors noted the presence of metastable assemblies that could not be explained by an aggregation mechanism based on a single pathway. To explain their findings, Korevaar *et al.* formulated a kinetic co-assembly model based on two different pathways representing metastable and equilibrium aggregates respectively. Simulations using realistic rate constants revealed that, counterintuitively, but in agreement with experiments, metastable aggregates were able to sequester monomers most efficiently in an intermediate temperature regime. This example clearly reveals that the presence of competing pathways can result in highly counterintuitive experimental findings, which can only be explained by mathematical modelling.

**Fig. 4:**
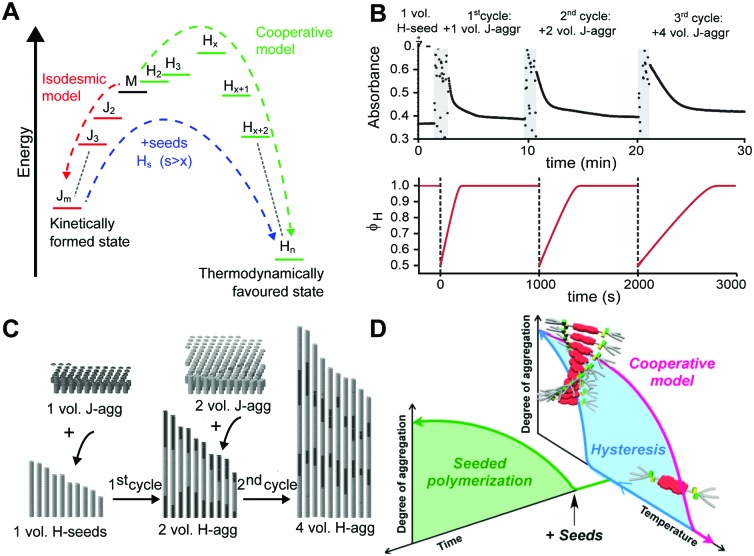
Living supramolecular polymerizations. (A) Energy landscape of two competing supramolecular polymerization pathways, where the formation of H-aggregates needs to overcome an energetic barrier corresponding to the appearance of critical nuclei. Adapted on permission of Macmillan Publishers Ltd: *Nat. Chem.*, ref. 27 copyright (2014). (B) Kinetics of the growth of the thermodynamically favored H-aggregates (top: experimental absorbance), adapted by permission from Macmillan Publishers Ltd: *Nat. Chem.*, ref. 27 copyright (2014); bottom: simulated fraction in H-aggregates, adapted from ref. 21, upon repeated addition of kinetically trapped off-pathway J-aggregates to initial H-seeds. (C) Snapshots of stochastic simulations of such a living supramolecular polymerization illustrating the block-wise growth. Adapted from ref. 21. (D) A living supramolecular polymerization obtained by the addition of seeds to kinetically trapped monomers in a thermal hysteresis. Reprinted with permission from ref. 28 Copyright 2015 American Chemical Society.

Where competition between the formation of thermodynamically stable (on-pathway) assemblies and metastable (off-pathway) assemblies may strongly affect the self-assembly dynamics by transient buffering of the available free monomers,^22^,^24^ this effect is enhanced in case of off-pathway aggregates become kinetically trapped. Takeuchi *et al.* cleverly exploited this phenomenon to engineer a living supramolecular polymerization.^27^ The authors designed a porphyrin dye, which can aggregate into thermodynamically stable H-aggregates *via* a cooperative mechanism as well as assemble into kinetically controlled J-aggregates *via* an isodesmic mechanism. Upon cooling a solution of porphyrin monomers, only kinetically trapped J-aggregates were formed. Conversion of these J-aggregates into thermodynamically stable H-aggregates was only initiated by the addition of H-aggregate seeds, *i.e.* separately prepared oligomers. This transformation corresponds to the energetic landscape consisting of two supramolecular polymerization pathways as illustrated in Fig. 4A, where the off-pathway J-aggregates form a kinetically trapped state, sequestering free monomers from solution, thereby hampering spontaneous nucleation of the thermodynamically favored H-aggregates. Despite the fact that the polymerization is non-covalent, the reaction kinetics are analogous to that of conventional chain growth polymerization, yielding supramolecular polymers with controlled length and narrow dispersity. Repeated addition of stock solution of J-aggregates resulted in further elongation of the H-aggregates, reducing the growth rate by a factor half in each step due to the dilution of the initial seeds (Fig. 4B top), while maintaining a low dispersity. Recently, Markvoort *et al.* used stochastic simulations to model such a stepwise living supramolecular polymerization process (Fig. 4B bottom), corroborating the living characteristics of such a mechanism (Fig. 4C).^21^

Later, Würthner *et al.*^28^ showed that such seeded living supramolecular polymerizations are not limited to situations where off-pathway aggregates sequester free monomers. The authors designed a perylene bisimide based molecule that in isolation preferentially adopts an aggregation-incompetent conformation *via* intramolecular hydrogen bonding. Upon cooling a monomeric solution, a kinetically trapped state could thus be formed in which the monomer is divided into predominantly aggregation-incompetent and a small fraction of aggregation-competent monomers. The resulting low concentration of aggregation-competent monomers retarded the spontaneous nucleation, leading to lag times up to more than an hour. The unique kinetics in this nucleation process was corroborated by characterization of the hysteresis. Addition of preassembled supramolecular seeds to the kinetically trapped state in such a hysteresis loop (Fig. 4D) led to the seeded formation of thermodynamically stable aggregates with low dispersity.

Another example where monomers are kinetically trapped in a non-aggregating form *via* intramolecular hydrogen bonds has been reported by Aida *et al.*^29^ They designed corannulene derivatives which adopt a cage-like closed conformation *via* intramolecular hydrogen bonding of five amide units. Though these intramolecular H-bonds are less robust than their intermolecular counterparts in the polymer chain, they again conformationally restrict the monomers from spontaneous polymerization. Cooperative aggregation was induced by mixing in a monomer variant with *N*-methylated amide units, lacking the capacity for intramolecular H-bonding. Serving as a proton acceptor for H-bonding, these initiator molecules lower the energy of the transition state for unfolding the regular monomers. The extraordinary stability due to the five-fold hydrogen bonds between consecutive monomers does not only give these initiator end-capped polymers a true living character, it also causes the formation of perfectly homochiral aggregates when assembling chiral variants of their monomers, something that was exploited to perform an unprecedented optical resolution of a racemic mixture using a chiral initiator molecule.

In concluding this section, we have seen that kinetic studies of supramolecular polymers are important to understand the assembly mechanism, even at thermodynamic equilibrium. Traditional tools pioneered in chemical kinetics in the 1950's, such as stopped-flow (dilution/co-solvation) or temperature-jump, are increasingly used to study supramolecular polymerization. Using these methods, many previously undetected metastable states and off-pathway processes have been exposed in the recent years. In addition, pathway selection has led to superior control over supramolecular structure and dispersity. Specifically, kinetic trapping (#2, Fig. 1A) has allowed for more monodisperse supramolecular polymers (dispersity close to 1.0), whereas at thermodynamic equilibrium (#1, Fig. 1A) the dispersity tends towards 2.

## 2. Methods to reach non-dissipative non-equilibrium states (#2, #3)

In this section, we focus on practical preparation methodologies by which non-dissipative non-equilibrium states #2 and #3, can be selectively reached or avoided along the assembly pathway. We selected examples where the preparation protocol leads to structurally different aggregates from the same building blocks under the same final experimental conditions, that is, solvent composition, temperature, pH, anionic strength *etc.* In other words, the final energy landscape is fixed, as a function of molecular design and final conditions, while the outcome of the aggregation process depends on the path by which monomers self-assemble into aggregates. Recall that for equilibrium states (#1, Fig. 1A), the final outcome is independent of the preparation procedure, but is only dictated by the final conditions.

### Modulating temperature

**Fig. 5:**
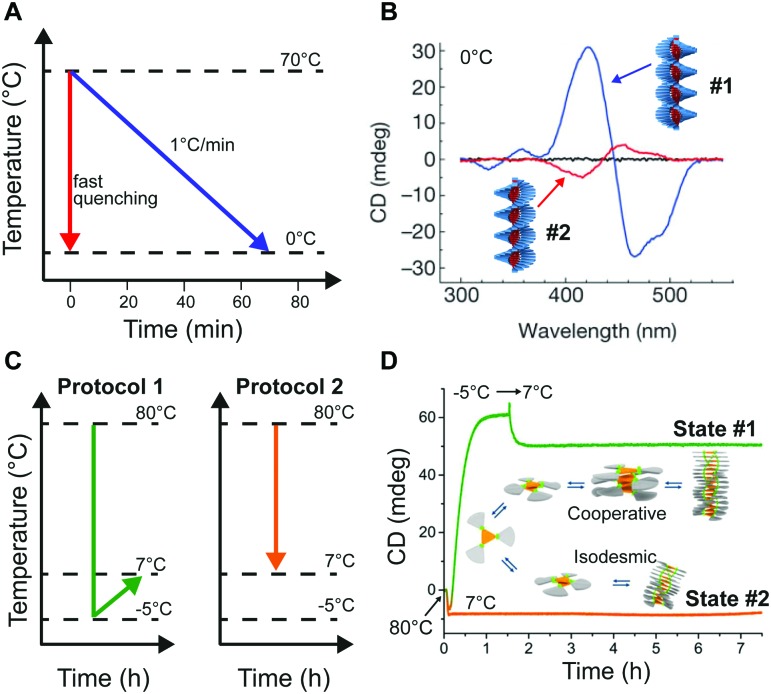
Modulating temperature to control self-assembly. (A) Schematic representation of different cooling rates from the molecularly dissolved state at 70 °C in MCH to the self-assembled state at 0 °C. (B) CD spectra of the thermodynamic (#1, blue line) and kinetic (#2, red line) *S*-OPV assemblies formed respectively by slow cooling (1 °C min^–1^) and fast quenching. Adapted from ref. 24 with permission from Nature Publishing Group, copyright 2012. (C) Different cooling protocols. (D) Two different CBT assemblies (#1 or #2) selectively obtained by using the cooling protocols reported in (C). The self-assembly process was monitored over time by following the CD signal at 490 nm. Adapted from ref. 30 with permission from American Chemical Society, copyright 2016.

Self-assembly in non-aqueous media is usually an enthalpy driven exothermic process, which is enhanced at lower temperature. As a consequence, a common methodology to induce aggregation is cooling down a molecularly dissolved solution of the building blocks. Both the cooling rate, as well as the shape of the heating/cooling profiles can be modulated to selectively obtain different assemblies, while targeting the same final temperature. As an example, Meijer and co-workers^18^,^24^ showed that upon slow cooling (1 °C min^–1^) a solution of a *S*-OPV from the molecularly dissolved state (70 °C in MCH) to 0 °C, the exclusive formation of the most stable left-handed helical assemblies was observed (equilibrium state, #1 in Fig. 1). However, fast quenching of the same solution yielded non-equilibrium kinetically trapped aggregates (state #2 in Fig. 1A) with right-handed helicity, see Fig. 5A and B. When heated to 25 °C the right-handed aggregates slowly converted to the thermodynamic ones, *i.e.* they are metastable (state #3 in Fig. 1A) at this temperature. In this case, as a consequence of the fast annealing, the system remained partially trapped in a local energy minimum, whereas the slow cooling allowed for complete equilibration. Recently Schmidt and Meijer^30^ showed that different self-assembled states of a chiral carbonyl-bridged triarylamine (CBT), with opposite CD spectra and different photophysical properties, can be selectively accessed under identical final conditions (7 °C, 50 μM CBT, in *o*-DCB solvent) depending on the thermal profile used during the assembling process (Fig. 5C and D). Namely, upon direct cooling from 80 °C (molecularly dissolved state) to 7 °C, a kinetically trapped product (state #2 in Fig. 1A) was formed *via* an isodesmic process, which remained stable for more than 5 h. On the other hand, the thermodynamically stable state at 7 °C could be obtained by the sequence 80 °C → –5 °C → 7 °C (*i.e.*, by first undercooling to –5 °C). The latter was formed *via* a cooperative polymerization mechanism. When the two kinds of assemblies were mixed in a 1 : 1 ratio, the kinetically trapped state rapidly converted in the thermodynamic one (<1 h). This, along with results from seeding experiments, demonstrated that the kinetically trapped aggregates are not able to self-nucleate due to high kinetic barriers, but can transform into the thermodynamic assemblies *via* a controlled aggregation only when nuclei are already preformed in solution. In addition, the process is highly enantioselective; in fact it only occurs if the kinetic and thermodynamic assemblies are made by building blocks with the same chirality. This example clearly shows how the distinction between kinetically trapped and metastable non-equilibrium states strongly depends on the presence of competitive polymerization mechanisms.

### Solvent processing

**Fig. 6:**
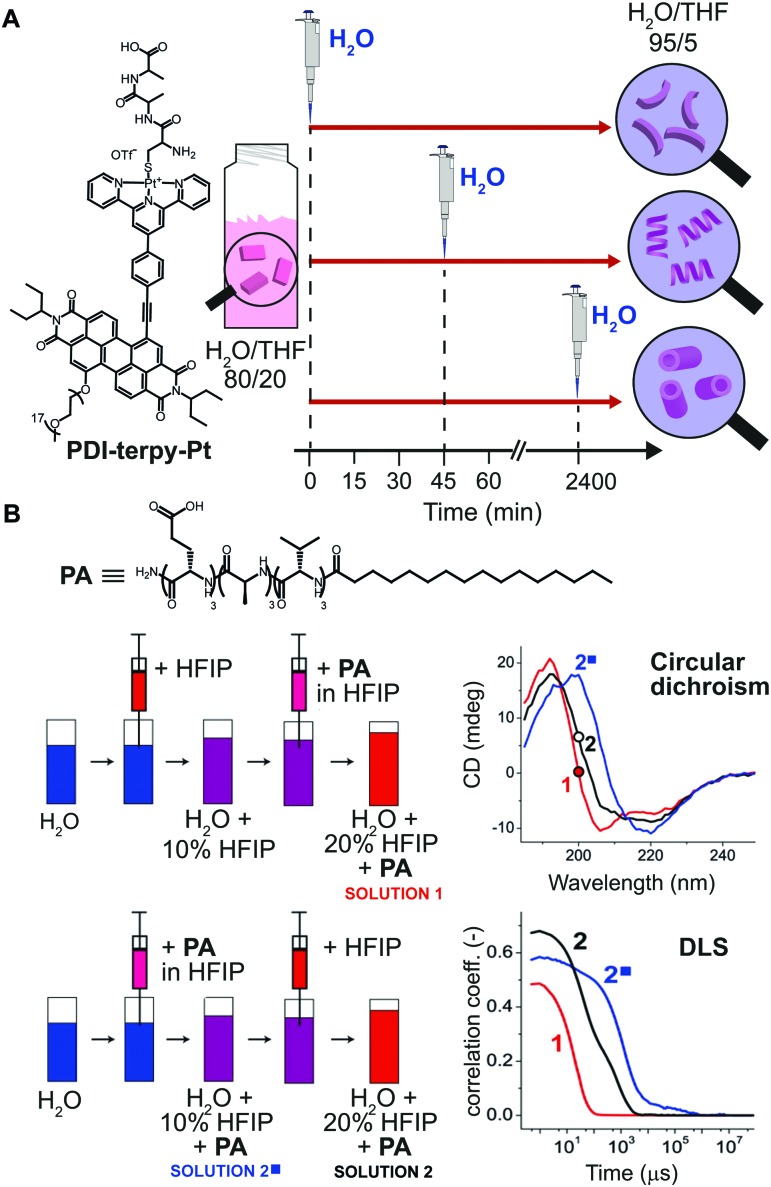
Pathway selection *via* solvent processing. (A) Schematic illustration of different kinetic assemblies of PDI-terpy-Pt obtained by diluting with water a water/THF = 80/20 mixture to 95/5 at different evolution times during self-assembly (0, 45, and 2400 min). (B) Two preparation protocols that differ by the order in which pure HFIP and the PA/HFIP stock solution were added to water were used to prepare two PA solutions with identical composition (50 μg mL^–1^ in 20% HFIP). Depending on the protocol, different PA assemblies were obtained as shown by CD and DLS spectra. Adapted from ref. 32 with permission from American Chemical Society, copyright 2014.

Another common method for inducing aggregation is to start with a molecularly dissolved solution of the building blocks in a good solvent, and changing the solvent composition by addition of a bad solvent, which enforces self-assembly of the molecular building blocks. In this case, experimental parameters, such as: concentration of the stock solutions, timing, and order of addition of the bad solvent, can be modulated to obtain structurally different assemblies. In fact, such parameters determine the actual conditions at which the nucleation takes place, and consequently its rate. As an example, Rybtchinski and co-workers^31^ reported on the pathway dependent self-assembly of a perylene diimide terpyridine platinum complex (PDI-terpy-Pt) based on a clever kinetic trapping (Fig. 6A). This system assembled into disordered curved fibers in water/THF = 95/5 (bad/good solvent respectively), with the CD spectrum showing a weak signal that did not evolve with time. On the other hand, at higher THF content (*e.g.* water/THF = 80/20) the CD signal of the as-prepared solutions strongly increased with time and the formation of straight fibers was observed after three days. In other words, at high water content the hydrophobic effect dominates, resulting into slow assembly/disassembly dynamics and strong kinetic trapping; whereas increasing the organic solvents attenuates the solvophobicity, yielding metastable assemblies that can equilibrate. The latter has been used to trap the metastable system at different evolution points. Namely, a water/THF = 80/20 solution of PDI-terpy-Pt was prepared and allowed to evolve for 0 min, 45 min or 2400 min, followed by dilution with water to 5% THF content (Fig. 6A). In spite of the same final composition, completely different assemblies were obtained (short curved fibers with 3.0 nm diameter, longer fibers with 3.2 nm diameter and nanotubes with 4.5 nm diameter, respectively), which did not interconvert into each other even after months (state #2 in Fig. 1A).

Another illustrative example of the importance of solvent processing has been reported by Stupp and co-workers^32^ in the self-assembly of a peptide amphiphile (PA). The latter is molecularly dissolved in the good solvent hexafluoroisopropanol (HFIP) and forms long cylindrical nanostructures in pure water, *i.e.* the bad solvent. Two PA solutions (50 μg mL^–1^) in 20% HFIP were prepared *via* two methods that differ by the order in which PA/HFIP stock solution and pure HFIP were added to water (solutions 1 and 2 in Fig. 6B). Albeit both solutions had the same final composition, *i.e.*, same PA concentration and HFIP content, clear differences in the molecular organization (β-sheet *vs.* random coil, shown by CD) and aggregate size (by DLS) were observed. In this case, the different outcome can be attributed to the primary nucleation step that is slower when it occurs at larger content of the good solvent (HFIP).

### Order of addition

**Fig. 7:**
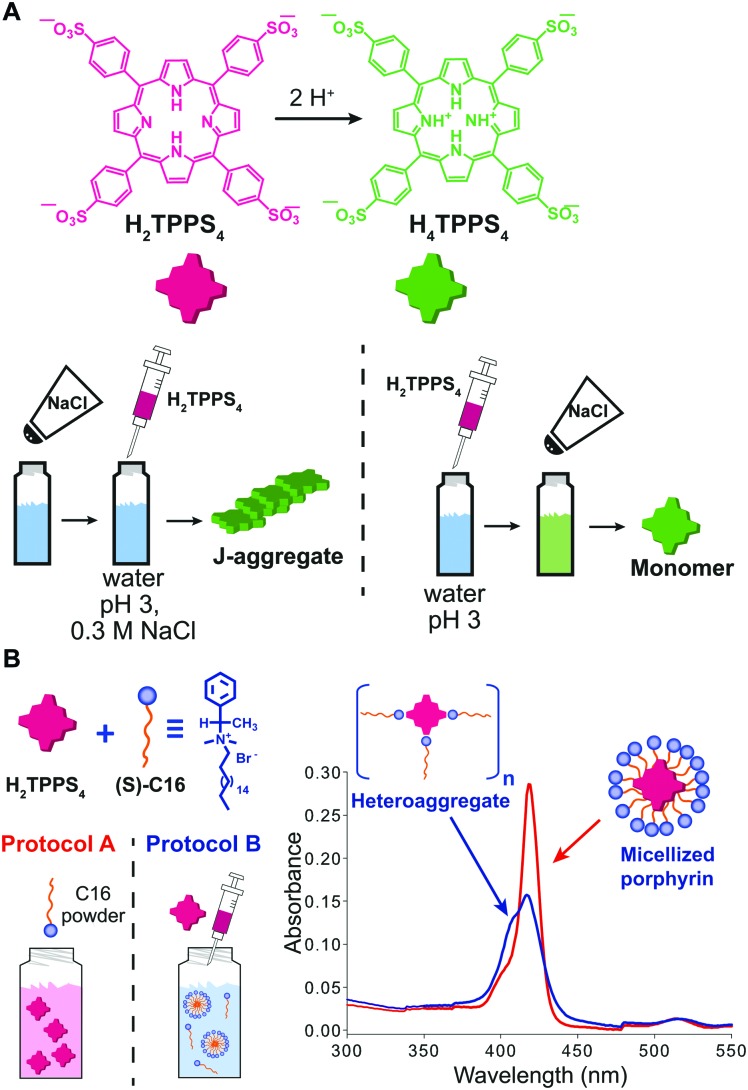
Effect of the order of addition on self-assembly. (A) Structure of H_2_TPPS_4_ and of its protonated form H_4_TPPS_4_, and schematic illustration of the effect of adding H_2_TPPS_4_ before or after NaCl to a pH 3 citrate buffer solution. (B) Two 1 μM H_2_TPPS_4_ solutions containing the chiral surfactant C16 at [C16]/[H_2_TPPS_4_] ratio 2500 : 1 were prepared either by dissolving the surfactant powder in a 1 μM H_2_TPPS_4_ solution (protocol A), or by adding a stock 1 mM porphyrin solution to the surfactant solution (protocol B), yielding respectively monomeric micellized porphyrin (red line in the UV-Vis spectrum), or surfactant/porphyrin heteroaggregates (blue line). Adapted from ref. 34 with permission from the American Chemical Society, copyright 2008.

When the onset of self-assembly is induced, for example, by the addition of protons to decrease pH and protonate the building blocks, or inorganic salts to screen aggregate charges, as well as in the case of multicomponent self-assembly (heteroaggregation), the hierarchical order of addition of the chemical species/constituents may strongly affect the outcome of the process. Purrello and Ribó extensively studied the self-assembly in water of ionic porphyrins, such as *meso*-(4-sulfonatophenyl)porphyrin (H_2_TPPS_4_), showing how it heavily depends on experimental conditions (concentration and ageing of stock solutions, pH, ionic strength, concentration of the working solution, *etc.*). For example, they found that the order of addition of protons and NaCl results in a clear “YES–NO” effect on the self-assembly of H_2_TPPS_4_ (at pH 3, [H_2_TPPS_4_] < 3.0 μM).^33^ Namely, when a stock aqueous solution of non-protonated H_2_TPPS_4_ was added to a pH 3 solution already containing NaCl, fast protonation and aggregation of the resulting H_4_TPPS_4_ occurred, leading to J-aggregates (the zwitterionic H_4_TPPS_4_, *i.e.* green in Fig. 7A, is the assembling unit). On the other hand, when NaCl was added afterwards, aggregation was not observed at all, resulting in monomeric protonated porphyrin that “survived” at conditions at which it is normally aggregated (Fig. 7A). This was ascribed to the presence of pre-nuclei in the stock solution of the non-protonated H_2_TPPS_4_ that, soon after being protonated, can rapidly elongate in the presence of salt (instead of dissolving otherwise). The latter was demonstrated by showing that upon working with a more diluted stock solution, J-aggregates were not formed at all, regardless of the order of addition of the components. Heteroaggregation of non-protonated anionic H_2_TPPS_4_ with the chiral cationic surfactant (*S*)-C16 (Fig. 7B) may yield either monomeric porphyrin included in surfactant micelles (CD silent) or to chiral surfactant/porphyrin heteroaggregates with a 3/1 stoichiometry (CD active), depending on the order of addition of the constituents (Fig. 7B).^34^ Namely, at surfactant concentrations above the critical micellar concentration (cmc), the dissolution of the surfactant powder in a dilute porphyrin solution yielded micellized monomeric porphyrin, whereas the addition of a concentrated porphyrin solution to a micellar surfactant solution led to the formation of heteroaggregates (Fig. 7B). So, once again, different assemblies could be selectively formed depending on the preparation protocol, even though the final solutions had identical composition. Remarkably, the two kinds of assemblies did not interconvert in each other even upon prolonged sonication.

### Multi-step non-covalent synthesis

**Fig. 8:**
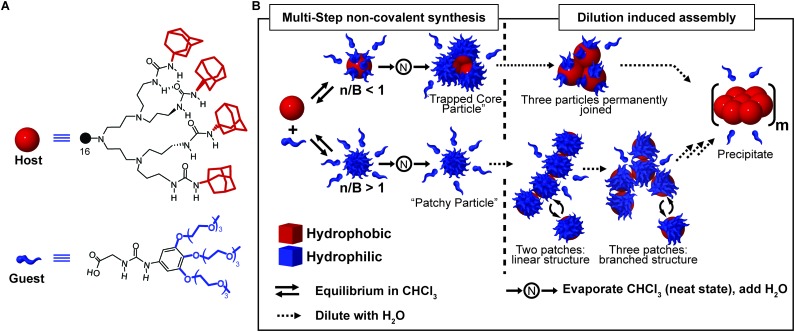
Multi-step non-covalent synthesis. (A) Molecular structure of the urea-adamantyl poly(propylene imine) dendrimeric host, and of the ureido acetic acid guest. (B) Host and guest are mixed in chloroform to form a pre-complex, which is later transferred to water *via* the neat state. Depending on the ratio of guest molecules and binding sites (n/B), either a small complex with three host molecules trapped in the core (for n/B < 1), or a patchy particle (for n/B > 1) are obtained. Dilution with water induces aggregation due to the dissociation of the host molecules which leaves hydrophobic patches exposed. Adapted from ref. 37 with permission from Nature Publishing Group, copyright 2009.

A powerful tool to obtain complex supramolecular structures, in which multicomponent assemblies are built by a sequence of processing steps, is to stabilize kinetically trapped intermediates (state #2 in Fig. 1A). An intriguing application is the preparation of supramolecular assemblies in solvents in which the individual components are not soluble. A well-known example is the formation of liposomes (archetypal non-equilibrium non-dissipative structures) implying the following steps: (i) formation of a lipid film from a good organic solvent (*e.g.* chloroform), (ii) hydration of the dry film to give a suspension of large multilamellar vesicles (LMV) with an onion-like structure, (iii) freeze–thaw cycles to reduce the lamellarity (large unilamellar vesicles, LUV, are formed), (iv) extrusion through a polycarbonate filter to select the size.^35^ One of us, reported on a multi-step approach to prepare dendrimer-based patchy nanoparticles in water.^36^,^37^ Such particles are formed by a hydrophobic dendritic host, the core, that can form guest–host interactions (salt bridges and H-bonding) at its periphery with water-soluble hydrophilic guest molecules, the latter decorating its surface (Fig. 8A). Because of the strong hydrophobicity, the host dendrimer cannot be dissolved in water, even in the presence of a large excess of guest molecules. However, if the guest–host complex is pre-formed in chloroform and dried, it can easily be transferred to water *via* the neat state, hence overcoming the barrier for the core solvation (Fig. 8B). In addition, the outcome of this stepwise approach greatly depends on the guest-to-host ratio used. When the number of guest molecules exceeds the number of binding sites, truly patchy particles consisting of a single host molecule with a corona of guest molecules are formed. On the other hand, if the number of guest molecules is lower than the binding sites at the host surface, kinetically trapped particles consisting of a cluster of host molecules (three on average) are formed (Fig. 8B). In addition, due to the reversible nature of the guest–host binding, the absorption equilibrium was exploited to control the average number of hydrophobic patches at the particle surface. Namely, at high concentrations, many guest molecules were absorbed, due to the effect of mass action, resulting in few randomly distributed patches, which in turn resulted in a weak net attraction between particles. On the other hand, dilution promoted dissociation of the guest molecules, thus leading to a larger number of hydrophobic domains exposed to water. As a consequence, the freely dispersed particles were destabilized, which promoted their aggregation (Fig. 8B). Increasing the concentration again after dilution did not lead back to the dispersed “patchy particle” (Fig. 8B), since the hydrophobic patches were too sticky.

In a beautiful example, Aida and co-workers used a stepwise strategy in which nanotubes of a fluorinated molecular graphene selectively nucleated and grew on pre-formed nanotubes of a non-fluorinated analogue, this last acting as seeds, thus creating a semiconducting supramolecular heterojunction.^38^

### Controlled diffusion

**Fig. 9:**
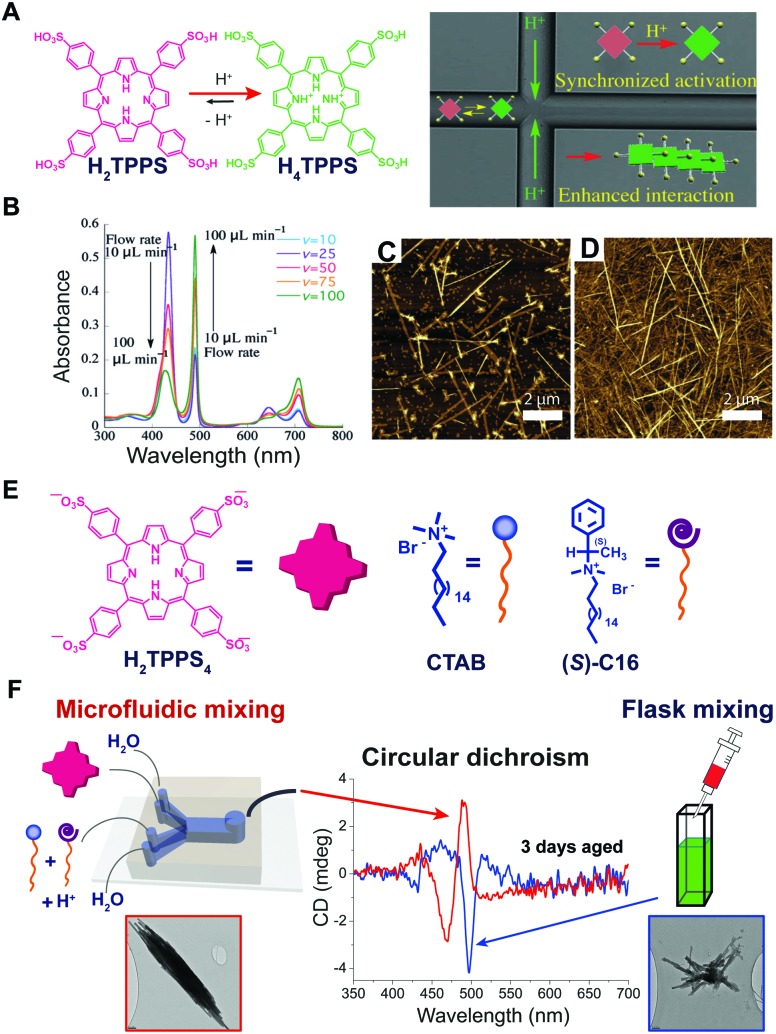
Effect of controlled diffusion on supramolecular polymerization. (A) Schematic representation of the concept of synchronized activation of H_2_TPPS (*i.e.*, protonation to H_4_TPPS) in a microfluidic chip yielding enhanced formation of J-aggregates. (B) UV-Vis spectra recorded for various flow rates (pH 3): J-aggregates absorb at 490 and 700 nm, monomeric H_4_TPPS at 434 nm. (C and D) AFM images of the J-aggregates prepared at pH 3.0 under microfluidic synthesis at flow rates 50 and 100 μL min^–1^ respectively. Adapted from ref. 39 (A–D) with permission from Wiley-VCH Verlag GmbH & Co., copyright 2015. (E) Chemical structure of H_2_TPPS_4_, CTAB and of the chiral inducer (*S*)-C16. (F) Effect of microfluidic *versus* flask mixing on the chirality of the formed H_2_TPPS_4_/CTAB heteroaggregates, *i.e.* on the efficiency of the chiral induction, and on the aggregate morphology shown in the TEM micrographs. Adapted from ref. 40 with permission from American Chemical Society, copyright 2016.

As we discussed above, some of the typical methods to induce self-assembly consist in adding a bad solvent, a co-assembling component, or other chemical species such as protons or salts, to a molecular dispersed solution of the building blocks. The latter is usually performed in a bulk reactor (*e.g.* a flask or a vial) followed by “uniform” mixing (*e.g.* manual shaking or vortex stirring). An underlying approximation is that the solution is homogeneous with each molecule experiencing the same experimental conditions on average, so that the onset of aggregation is a statistical process that occurs randomly in space and time. However, supramolecular chemists are aware of the role that local gradients in reactant concentration, or solvent composition, *e.g.* due to imperfect mixing, may have on the outcome of the self-assembly, sometimes rendering it scarcely reproducible. In particular, this happens when non-dissipative non-equilibrium structures (*i.e.*, states #2 or #3 in Fig. 1A) formed by nucleated polymerization are involved, and when molecular diffusion is commensurate with the nucleation rate. On the other hand, the careful control of reactants (or solvent) diffusion can be envisaged as a powerful tool for the selective preparation of different non-equilibrium structures using pathway selection. In this regard, the use of microfluidic mixing can be a valuable approach. In fact, due to the laminar flow conditions in the microchannel, the solutions mix only by molecular diffusion. By modulating parameters, such as total flow rate, flow focusing, and the chip geometry, it is possible to precisely define the reaction–diffusion zone, *i.e.*, the concentration gradients and solvent composition in space, and the interfaces at which nucleation occurs. Numata and co-workers^39^ used microfluidic conditions for the kinetically controlled preparation of porphyrin assemblies from nano to micro scale. For example, they found that the polymerization of H_2_TPPS to form J-aggregates, as triggered by its protonation, is strongly enhanced under microfluidic mixing, and occurs at final conditions at which it is not observed in a flask (Fig. 9A). In addition, the degree of aggregation and the (homogeneous) length of the assemblies could be nicely modulated by changing the flow rate (*i.e.* affecting the proton concentration profile), revealing an exquisite control over nucleation (Fig. 9B–D). In another example, the heteroaggregation of H_2_TPPS_4_ with cetyltrimethylammonium bromide (CTAB) was studied in the presence of small amounts of the chiral surfactant (*S*)-C16, as a chiral inducer (*i.e.* the “sergeant”), both under microfluidic and flask mixing.^40^ Flask synthesis produced metastable assemblies (state #3 in Fig. 1A) whose supramolecular chirality slowly evolved with time (3 days) to those always observed in the absence of the inducer. On the other hand, microfluidic mixing allowed for efficient chiral induction, even at very low concentration of the inducer. In addition a different morphology for the aggregates prepared by the two kinds of mixing was observed (Fig. 9F). Remarkably, only 20 milliseconds of controlled diffusion (*i.e.* the residence time of the solutions in the chip) at the very early stages of the assembly process (*i.e.* during nucleation) were enough to influence the expression of chirality in this evolving tri-component non-equilibrium non-dissipative system.

### Hydrodynamic fields

**Fig. 10:**
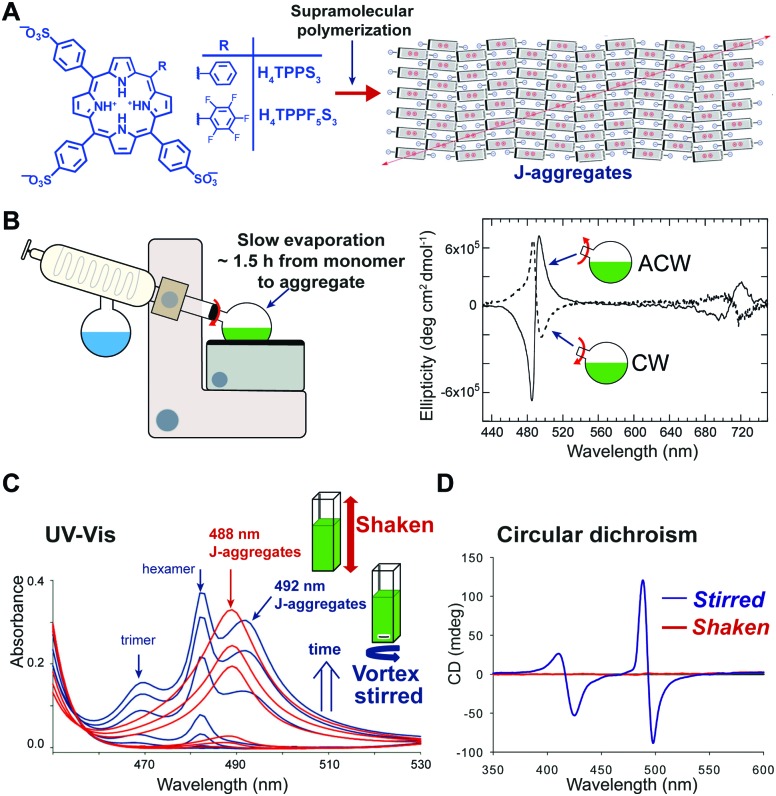
Pathway selection *via* hydrodynamic fields. (A) Diprotonated 4-sulfonatophenyl *meso*-substituted porphyrins self-assemble hierarchically to give J-aggregates. This aggregates are based on an intrinsically chiral 2D sheet structure, that depending on the porphyrin *meso*-substitution pattern, and on the preparation protocol can assemble further to give bi- or multi-layered structures (*cf.*ref. 41 and 44). Adapted from ref. 44 with permission from The Royal Society of Chemistry. (B) Chiral sign selection in H_4_TPPS_3_ J-aggregation depending on the direction of stirring during the rotary evaporation of diluted monomeric solutions to assembled ones. Adapted from ref. 42 with permission from The American Association for the Advancement of Science, copyright 2001. (C) Typical evolution of the aggregation of H_4_TPPF_5_S_3_ under shaking (uniform mixing) or magnetic stirring (imperfect mixing) conditions, as monitored by UV-Vis spectroscopy. Adapted from ref. 45 with permission from The Royal Society of Chemistry. (D) CD spectra of H_4_TPPF_5_S_3_ aggregates obtained under vigorous-shaking and magnetic stirring. Adapted from ref. 46 with permission from Wiley-VCH Verlag GmbH & Co., copyright 2012.

In some cases, hydrodynamic flows have been used as a tool to finely control the structure and supramolecular chirality of growing supramolecular assemblies allowing to obtain exquisite pathway selection. The effect of the hydrodynamic field can be mediated by different mechanisms^41^ including: (i) alignment/orientation of the anisotropic particles in the flow, (ii) deformation due to the torque generated by the gradient of flow velocity, and (iii) shear-induced breaking. It has to be noted that these effects are well-recognized in colloids chemistry, whereas in supramolecular chemistry they are often overlooked. In a seminal work, Ribò *et al.*^42^ demonstrated that vortex stirring during the aggregation of achiral diprotonated porphyrin H_4_TPPS_3_, promoted by slow rotary evaporation, yields chiral J-aggregates whose sign depends deterministically on the direction of rotation (Fig. 10A and B). More recently, Micali and co-workers^43^ reported an analogue effect of chiral sign selection in J-aggregates of H_4_TPPS_3_, by applying a combination of rotating and magnetic forces only at the very beginning (nucleation step) of the aggregation. Porphyrin J-aggregates are intrinsically chiral structures (*i.e.* with achiral monomers arranged to form a chiral crystalline cell), so the emergence of optical activity has to be ascribed to the formation of enantiomerically enriched mixtures of J-aggregates, so called scalemic mixtures.^44^ Vortex stirring during the nucleation stage can result in a small enantiomeric bias (*e.g.*, by a weak torque), which is then amplified in the elongation process resulting in a scalemic mixture. This corresponds to a scenario where even a tiny chiral perturbation is sufficient to induce chiral sign selection in the spontaneous mirror symmetry breaking. As another example,^45^ the self-assembly of a fluorinated porphyrin H_4_TPPF_5_S_3_ both in vortex stirred solutions and shaken solutions showed that structurally different J-aggregates can be selectively formed depending on the kind of mixing (Fig. 10C). This was ascribed to the alignment of the growing particles in the laminar flow generated by magnetic stirring in a square section-flask, reducing their lateral diffusion and thus inhibiting cluster-to-cluster aggregation mechanisms. Furthermore, aggregates obtained under gentle stirring showed an intense CD signal whose sign did not correlate with the stirring direction, whereas aggregates obtained under vigorous shaking were CD silent (Fig. 10D).^46^ A reasonable explanation is that under strong shaking a high number of chiral primary nuclei was formed, thus favoring a racemic situation, whereas, vortex-stirring favored the formation of a low number of nuclei most likely evolving to an enantioenriched mixture.^41^,^46^ Note that the latter is similar to the crystallization of achiral salts, like NaClO_3_, which under stirring can form scalemic mixtures of enantiopure crystals (*e.g.* Kondepudi crystallization scenario).

### Template assisted self-assembly

**Fig. 11:**
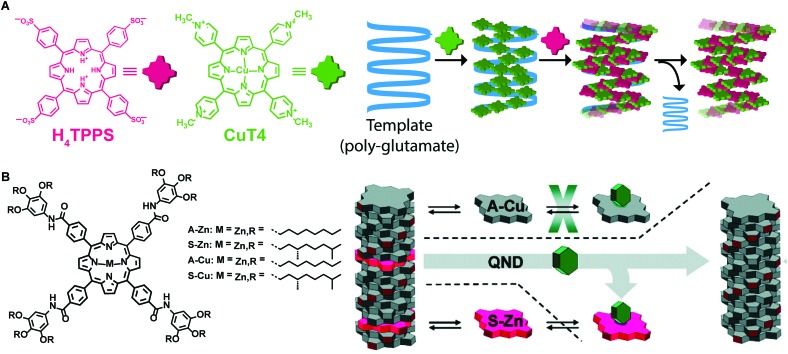
Template assisted self-assembly. (A) A tetracationic porphyrin (CuT4) is assembled on a polyglutamate template, afterwards, a tetraanionic porphyrin (H_4_TPPS_4_) is assembled in the pre-templated aggregates. Removing the template, the formed assemblies were retained. (B) Chiral porphyrin assemblies were formed *via* a “sergeant-and-soldier” strategy, removal of the sergeant Zn–porphyrin by axial ligation results in the kinetically trapped assemblies of Cu–porphyrin. Adapted from ref. 48 with permission from the American Chemical Society, copyright 2010.

Templating is another strategy that has been used to control morphology and molecular organization of non-dissipative non-equilibrium structures. The template guides the self-assembly of the molecular building blocks to form structures that otherwise would not be formed spontaneously without templating, thus decreasing the energy barrier for a specific pathway. Once the template is removed, the formed supramolecular structure is preserved, if it resides in a deep enough energy well (*i.e.*, if it is a state #2). One of the most common applications of templating is imprinting supramolecular chirality to assemblies formed from achiral building blocks. For example, Purrello and co-workers^47^ studied the aggregation of achiral water-soluble porphyrins onto oppositely charged polymeric chiral templates (*e.g.* α-helix forming polypeptides). They reported on a tri-component supramolecular system prepared step-wise by assembling a tetra-cationic porphyrin on an anionic polyglutamate template, followed by the addition of tetra-anionic porphyrin, which assembled on the “pre-templated” aggregates (Fig. 11A). Remarkably, upon removing the template, the supramolecular chirality of the porphyrin assemblies was retained because of their kinetic stability due to slow dynamics in water, resulting in the storage of the imprinted chiral information. In addition, the porphyrin chiral assemblies acted themselves as templates when fresh monomeric porphyrins were added. Meijer and co-workers^48^ cleverly combined chiral amplification *via* a “sergeant-and-soldiers” approach with selective removal of the “sergeant” to obtain assemblies with defined supramolecular chirality from an achiral Cu–porphyrin (Fig. 11B). Namely, a chiral Zn–porphyrin, the “sergeant”, was co-assembled with an achiral Cu–porphyrin, “the soldier”, thus transferring its own chirality to the whole aggregate. Afterwards, the “sergeant” was removed from the binary assemblies by Zn axial ligation with a Lewis base, resulting in kinetically stable assemblies of Cu–porphyrin with desired chirality.

### Chemical fuels

**Fig. 12:**
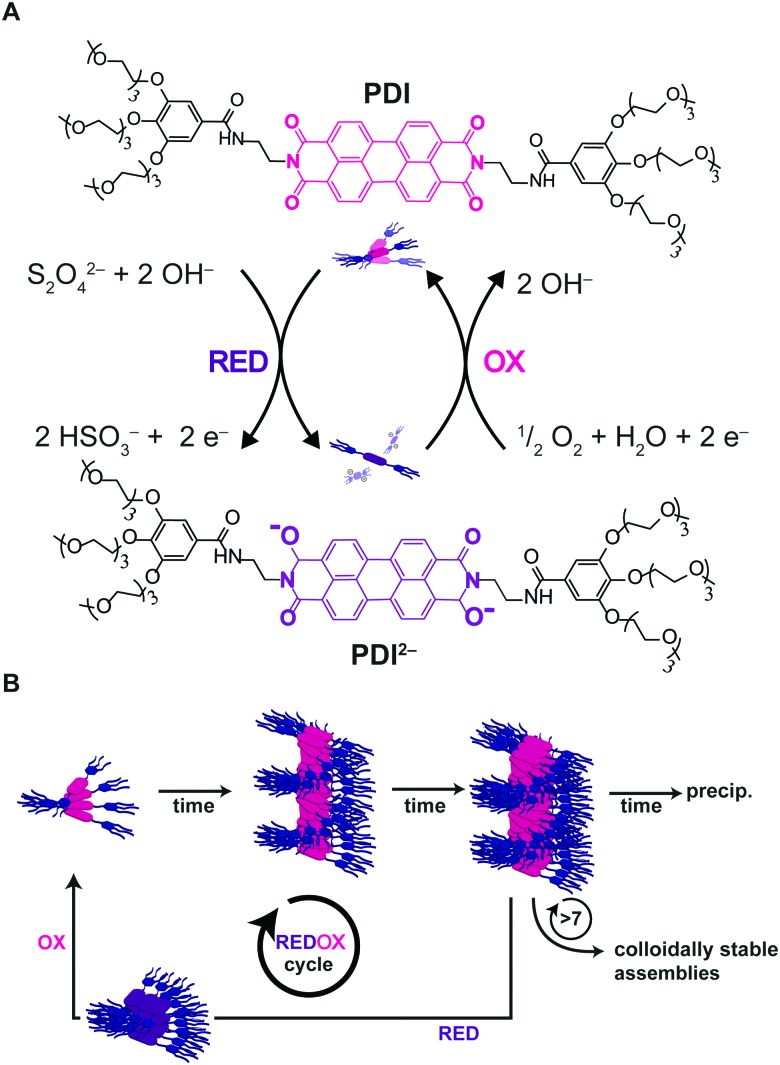
Chemical fuels. (A) PDI was reduced upon addition of dithionite to the dianion PDI^2–^, upon exposure to air PDI^2–^ is oxidized back to the neutral PDI. (B) The number and frequency of redox cycles will affect the outcome of the assemblies due to progressive changes in the assemblies after each redox cycle. Adapted from ref. 49 with permission from The Royal Society of Chemistry.

Recently, Hermans and co-workers^49^ reported on a novel approach to achieve pathway selection in the self-assembly of a perylenediimide derivative (PDI) in water, which is based on the use of two redox agents as chemical fuels to drive assembly and disassembly cycles. The strong aggregation of PDI in water is dominated by the hydrophobic effect, resulting in high kinetic barriers and slow exchange dynamics. Heating/cooling cycles to overcome such barriers could not be used, since heating promoted further aggregation and precipitation, due to the hydrophobic effect. So, the authors exploited the reversible reduction of PDI by dithionite (S_2_O_4_^2–^), leading the dianion PDI^2–^, as a way to disassemble the aggregates (*i.e.* due to electrostatic repulsion). Afterwards, the dianion could be oxidized back upon exposure to air (O_2_) giving neutral PDI that as soon as formed, assembled back by a hierarchical nucleated polymerization (Fig. 12A and B). Interestingly, progressive changes in the structure and packing of the assemblies (*i.e.* changes in pathway selection) were observed after each redox cycle. In particular, not only the number, but also the frequency of the redox cycles, was found to determine the final structure of the assemblies (Fig. 12B). In other words, there is a “memory effect” propagating along the cycles that has been ascribed to the incomplete disassembly of PDI^2–^, causing trace assemblies to remain that preserve the structure they had in the previous cycle. In short, the use of chemical fuels to drive assembly/disassembly cycles can be a valid approach to control pathway selection, allowing the system to circumvent strong kinetic trapping by overcoming assembly barriers.

## Applications and outlook

A wide number of preparation tools have been used in recent years to control the outcome of supramolecular polymerization. In addition, the mechanistic understanding of many such systems has progressed due to kinetic models that consider parallel assembly pathways. The key lesson learned so far is that experimental conditions have to be monitored and controlled with high precision. For example, a slightly higher temperature in the laboratory or a different grade (purity) solvent might change the assembly outcome of the system under study. This realization has also permeated into industry, where companies are now connecting their equipment to the cloud to monitor and control exact conditions.^50^

In academia, the rise of easy to use and cheap platforms like Arduino or Raspberry Pi, are allowing researchers to couple sensors and equipment together. In this way, a syringe pump can for example be coupled to a UV-Vis spectrometer, to add an aliquot of solution when a certain absorbance is reached. Of course, the latter approaches have to be extended to control all relevant experimental conditions (*e.g.*, temperature, solvent composition, order of addition, *etc.*). Combinations of well-programed temporal or even simultaneous actions can then be envisaged. Overall, better control will allow novel non-covalent syntheses to be designed and performed, leading to a wide range of distinct self-assembled structures that are based on the same building blocks. Controlling the shape, molecular organization, chirality, and dispersity of self-assembled structures is crucial for applications *e.g.* in supramolecular materials, energy conversion and biomedicine.^4^
